# Assessing ambulance staff attitudes toward mental health conditions: translation and psychometric evaluation of the medical condition regard scale among ambulance staff

**DOI:** 10.1186/s40359-024-02312-5

**Published:** 2024-12-27

**Authors:** Kristin Häikiö, Carl Robert Christiansen, Rune Kveen, Eva Marie Engebakken Flaathen, Milada Hagen

**Affiliations:** Faculty of Health Sciences, Department of Nursing and Health Promotion, Oslo Metropolitan University, Oslo, Norway

**Keywords:** Ambulances, Paramedic, Mental health, Attitude, Psychometrics, Mental disorders, Scandinavian and nordic countries, Emergencies

## Abstract

**Introduction:**

Ambulance staff play a crucial role in responding to mental health crises. However, negative regard toward patients with mental health conditions can hinder care. The Medical Condition Regard Scale (MCRS) assesses regards or attitudes but has not previously been validated for educated ambulance staff and has never been translated into Norwegian. This study aims to translate the instrument into Norwegian, test it on a population of ambulance staff, explore the psychometric properties of the Norwegian version, and measure regard for patients with psychosis.

**Method:**

The MCRS is an 11-item instrument with a Likert scale of 1–6. Possible sum scores range from 11 to 66 (higher score = more positive regards). We chose “psychosis” as the condition to investigate. Translation followed eight steps: (1) preparation, (2) forward translation, (3) backward translation, (4) first expert panel review, (5) harmonisation, (6) cognitive debriefing, (7) second expert panel review, and (8) writing of the final version. The instrument was tested and re-tested regarding the condition “psychosis” on a representative sample of 114 Norwegian ambulance staff in 2023, with a temporal gap of one month. We explored item scores and distribution, as well as floor and ceiling effects. We tested the internal consistency of the items using Cronbach’s Alpha and consistency in answers over time (test and re-test) using the Paired Sample-T test. We used factor analyses to explore the inter-item relationships of the items.

**Results:**

The 114 participants had a mean sum score of 47, which is mid-range. The scale has a ceiling effect on five items, which was not described in detail earlier. Two items regarding the monetary spending on patients with the given condition had the largest ceiling effects. However, the Norwegian translation showed adequate internal consistency (Cronbach’s Alpha = 0.82) and is reliable over time. Test and re-test showed no significant differences in the scale’s total score (Paired sample T-test, *p* > 0.05). Exploratory and confirmatory factor analyses indicate that the scale should be used as a one-dimensional instrument in a Norwegian setting in ambulance staff populations.

**Conclusion:**

The Norwegian translation of the MCRS is a reliable instrument for ambulance staff measuring medical condition regards. However, the ceiling effect limits the ability to discern differences among high-scoring individuals. Ambulance staff’s regard for patients with psychosis is medium positive (mid-range level), but slightly more positive than what is reported in the international literature regarding patients with mental health issues.

## Background

Deinstitutionalisation and pressures on mental health services globally have caused a shift of care from inpatient healthcare services to community-based settings and law enforcement officers [1]. Consequently, emergency services worldwide report rising numbers of emergency assignments to people experiencing mental health crises outside of hospitals, whicht usually make up around one-third of medical emergency calls [2]. Consequently, ambulance staff often responds to mental health crises, including psychosis [3, 4]. Despite established relationships with community mental health services, patients with mental health issues frequently rely on emergency medical services during off-hours [5, 6]. In acute mental health crises, ambulance staff are the first healthcare providers on the scene. Their attitudes and approach can either facilitate or hinder patients’ access to mental health care and medical assessment [7, 8]. and a 20-year mortality gap for men and 15 years for women have been reported for people living in high-income countries with a mental illness, Scandinavian countries included [9]. 

The ethical principle of impartiality requires healthcare professionals to treat patients regardless of the patient’s background [10, 11] and regardless of disease, disability or social standing. However, some healthcare professionals, including ambulance staff, exhibit stigmatising attitudes toward people with mental health conditions [12]. A study from Australia found that ambulance staff held more stigmatised opinions and attitudes towards patients with psychosis than towards people with depression and were less willing to spend time with patients with psychosis [13]. Such attitudes may discourage help-seeking among patients [12] and influence healthcare workers to pursue less aggressive treatment and more avoidant clinical behaviours [14]. To address this, the Medical Condition Regard Scale (MCRS) was developed by Christison, Haviland and Riggs et al. [14]. 

The MCRS assesses biases, emotions, and expectations related to medical conditions among students and caregivers. Although validated for assessing regard across various medical conditions [14], the MCRS has not yet been translated to Norwegian or validated in a Norwegian ambulance staff population. Our study aims to fill this gap by translating MCRS into Norwegian, validating it for use in prehospital settings, exploring the psychometric properties of the Norwegian version, and exploring the medical regard of Norwegian ambulance staff towards patients with psychosis. The condition “psychosis” was chosen because it is a more specific condition than the general term “mental health”. To validate the MCRS we considered it necessary to choose a more specific condition than the term “mental health”. The latter includes a broad spectrum of conditions with a large variation of signs and symptoms, while the condition “psychosis” is a more specific condition relevant for emergency situations involving ambulance staff.

## Methods

### The context

The Norwegian ambulance services operate as departments under 18 of the 20 public hospital trusts (HT). These HT are state-owned and organised in four regional health trusts (RHT), covering both rural and urban areas. According to Statistics Norway, the total number of ambulance staff is 5355 (private communication, October 4th, 2023). Most ambulance staff in Norway are either Ambulance Service Technicians (AST) with or without an additional paramedicine certificate (*n* = 3649, 68.1%) or hold a bachelor’s degree in Paramedicine (BP) (*n* = 450, 8.4%) or Nursing Science (BN) (*n* = 1256, 23.5%). Consequently, about 32% of ambulance staff in Norway hold either a BP or BN. In this study, we classified those with a completed bachelor’s degree in nursing or paramedic science as ‘higher education,’ while those without such degrees fall under ‘lower education.’ We refer to the combined group of AST, BP, and BN working clinically in ambulance services as ‘ambulance staff’. Typically, two persons staff each ambulance, and although they can seek advice from others, they usually make independent decisions regarding medical assessments and further transport.

### Translation and cultural adaptation of the medical condition regard scale (MCRS)

The MCRS instrument consists of 11 items, each to be answered on a Likert Scale ranging from 1 (strongly disagree) to 6 (strongly agree). The total sum score can range from 11 to 66, with higher scores reflecting more positive regard. During preparation, the original developer [14] granted permission for us to translate the MCRS. Following this, we established an expert panel. The panel included bilingual paramedics, translators, linguists, and researchers proficient in English. The translation process followed established guidelines [15, 16]. It involved eight steps: (1) preparation, (2) forward translation, (3) backward translation, (4) expert panel reviews, (5) harmonisation, (6) cognitive debriefing, and 8. final version writing.

Notably, items two and seven required cultural adaptation due to differences between private health insurance-based systems (original version) and Norway’s publicly financed healthcare system. For instance, item two changed from ‘Insurance plans should cover patients like this to the same degree as other conditions’ to ‘Patients like this should receive as much help from the public healthcare service as patients with other conditions’ in Norwegian. Similarly, item seven was modified to address public resources rather than medical dollars.

The cognitive debriefing tested the MCRS instrument for regards toward patients with psychosis (See Table 1 for all items in the MCRS instrument) using a focus group interview with four ambulance staff with varied work experience (2–30 years) and different levels of education (AST’s, BNs and BPs). KH and EMEF, both with previous experience with focus group interviews, facilitated the cognitive debriefing process and took notes of the discussions. The focus group participants highlighted several important points. First, they pointed out the need for a neutral option on the Likert scale. Second, they agreed that ambulance staff often lack knowledge of patients’ diagnoses, making questions about conditions like psychosis potentially confusing. Third, item five caused confusion due to ambiguity about whether annoyance was related to the patients or to the challenging situations. Fourth, item six inaccurately assumed that ambulance staff sleep during night shifts, leading to reduced trust in the researcher’s understanding of their work. Finally, item eight raised uncertainty about whether it measured personal feelings or practical difficulties in managing ambulance assignments.

We addressed item six based on focus group discussions. Additionally, we considered minor linguistic adjustments for items two, four, nine, and ten, incorporating some changes that made the Norwegian version closer to the original version. However, for items five and eight, we kept the wording in the Norwegian version since potential confusion about the item was also present in the original version. The final Norwegian version is shown in Table 1.

**Table 1:** The Norwegian version and the original English version of the MCRS

The Norwegian version of the MCRS	English (original) version of the MRCS
ANSEELSESSKALA FOR MEDISINSKE TILSTANDER Bruk følgende skala til å rangere hvor enig eller uenig du er i påstandene om pasienter med _______________ A = Svært uenig B = Uenig C = Ikke helt sikker, men trolig uenig D = Ikke helt sikker, men trolig enig E = Enig F = Svært enig Angående pasienter med: _________________	THE MEDICAL CONDITION REGARD SCALE Use the scale below to rate your degree of agreement or disagreement with each of the following items regarding patients with _________________________________. A = Strongly disagree B = Disagree C = Not sure, but probably disagree D = Not sure, but probably agree E = Agree F = Strongly agree Regarding patients with _________________________:
1. Å arbeide med pasienter som dette er tilfredsstillende.	1. Working with patients like this is satisfying.
2. Pasienter som dette bør få like mye hjelp av helsevesenet, som pasienter med andre tilstander.	2. Insurance plans should cover patients like this to the same degree that they cover patients with other conditions.
3. Det er lite jeg kan gjøre for å hjelpe pasienter som dette.	3. There is little I can do to help patients like this.
4. Jeg opplever spesiell medfølelse overfor pasienter som dette.	4. I feel especially compassionate toward patients like this.
5. Pasienter som dette irriterer meg.	5. Patients like this irritate me.
6. Det ville ikke plage meg å måtte hjelpe pasienter som dette på natten.	6. I wouldn’t mind getting up on call nights to care for patients like this.
7. Å behandle pasienter som dette er bortkastede offentlige midler.	7. Treating patients like this is a waste of medical dollars.
8. Pasienter som dette er spesielt vanskelige for meg å jobbe med.	8. Patients like this are particularly difficult for me to work with.
9. Jeg klarer vanligvis å gjøre noe som får pasienter som dette til å føle seg bedre.	9. I can usually find something that helps patients like this feel better.
10. Jeg trives med å vie ekstra tid på pasienter som dette.	10. I enjoy giving extra time to patients like this.
11. Jeg foretrekker å ikke jobbe med pasienter som dette.	11. I prefer not to work with patients like this.

### Survey data collection

The psychometric testing in this study was conducted on the 11-item final Norwegian version (see Table 1), with items rated on a Likert scale ranging from 1 (strongly disagree) to 6 (strongly agree). For the final sum score, higher scores indicated more positive regard. In addition, we collected information about participants’ age, sex, years of experience from working in ambulance services, educational level, and in which region they were employed.

We collected data in two waves to do a test and re-test. The first data collection (D1) was realised between the 6th and 23rd of September 2023. The second data collection (D2) was carried out between the 24th of October and the 12th of November 2023. An open-access link to an online self-administered survey was used to gather data. We published invitations and links to participation in the study in relevant groups and pages on Facebook. Invitations to participate in the survey were also sent by e-mail to ambulance staff in the authors’ network. Additionally, we asked paramedic colleagues to share the invitation using their networks. An information letter was linked to the survey; completing and submitting the questionnaires was considered consent to participate.

Participation was voluntary and could be done anonymously. In D1, we asked participants if they would allow us to contact them again after a few weeks for a follow-up survey. Those who accepted provided their email address and received the same survey on the 24th of October, 2023. The survey was open for three weeks; three reminders were sent.

### Recruitment strategy and selection bias limitations

We decided that the level of education (high or low) and the geographical location of respondents, primarily place of work (four regions), were the two most important characteristics describing ambulance staff in Norway. Education level is found to be associated with healthcare workers’ attitudes toward patient groups [17, 18]. Furthermore, the culture of organisations and workplaces is believed to adapt to changing environmental requirements, and the literature suggests a reciprocal relationship exists between culture and attitudes [19, 20]. Consequently, we believe that cultures and attitudes may differ between geographic regions. Based on these two characteristics (level of education and geographic region), we could use data from Statistics Norway regarding education and geographic region to describe the distribution of eight possible combinations of education and geographical location. Monitoring our ongoing recruitment and study sample regarding these categories enabled us to monitor the proportions of included individuals for given geographical areas and levels of education. This enabled us to strategically invite more respondents from the categories with an insufficient number of participants and increase our chances of having a representative sample. KH monitored the dataset and commented on Facebook to encourage ambulance staff from regions with a low number of participants and higher or lower levels of education to volunteer for the study.

## Statistical analyses

### Sample size considerations

Power calculations, based on the number of items and the intention to perform test-retest and regression modelling, indicated that 65 completed responses would be sufficient. However, more data would increase the statistical power and thus, the precision of our estimates. Consequently, we chose to use all available respondents in our final analysis.

To evaluate the psychometric properties of the MCRS we tested the reliability of the scale using a test-retest approach. The validity and internal consistency of the items were assessed using Cronbach’s alpha [18] and by describing the effects of ceiling and flooring. Cronbach’s alpha > 0.7 was considered acceptable. Statistical analyses were conducted by KH and MH using the software IBM SPSS statistics version 28 [21]. 

### Data cleaning and sensitivity analyses

We started with exploring the datasets D1 and D2 (test and re-test), checking for errors, checking the distributions of variables, as well as computing the sum score and the scores for each item. We discovered that 41 respondents had given more than one response on the Likert Scale, usually on one or two of the 11 items (one respondent had given three answers on one item), leading to ambiguity concerning which of the given responses should be included in the analyses. For this reason, we conducted a sensitivity analysis for each data collection (D1 and D2) using two different datasets for each of the data collections – one where we kept the most positive scores (positive regards) on each item (HiD1 and HiD2) and one where we kept the least positive scores (least positive regards) on each item (LoD1 and LoD2). Consequently, we present results from analyses of four datasets (HiD1/HiD2 and LoD1/LoD2).

### Reliability and validity assessments

To investigate possible floor and ceiling effects, we computed the sum scores and the scores for each item as well as the number (%) of responses.

By the convention in the field, we considered the ceiling or floor effects to be present if more than 15% of the respondents answered the highest or lowest score on each of the included items [22]. 

To test the reliability of the scale, we investigated the internal consistency of the items in the scale by calculating Cronbach’s Alpha. According to Tavakol and Dennick et al. [23], values between 0.7 and 0.9 are considered acceptable, while values above 0.9 can indicate that the items are too similar. The correlation between answers given during the test-retest assessment periods was investigated using the parametric Paired sample T-test. We report mean values, 95% confidence intervals and two-sided *p*-values. Following conventions, *p*-values are considered statistically significant if lower than 0.05. A sensitivity analysis was done using the non-parametric Wilcoxon signed ranks test.

## Results

In the first data collection (D1) we received 266 completed questionnaires. Of those, 210 participants were invited to answer the survey for the second time. We received 114 completed questionnaires from the second data collection (D2).

### Description of the sample

As there is only a limited amount of statistics on ambulance staff in Norway, available variables (age groups, sex, educational level, region of employment) provided by Statistics Norway (private communication, 02.10.2023) were used to assess the representativeness of our sample for the population of ambulance staff working clinically in Norwegian ambulance services. Of the 114 respondents who answered the questionnaire twice, the collected background variables had a similar distribution in our sample as in the general population of ambulance staff (see Table 2). However, respondents in our sample were slightly younger, slightly more often employed in the Central Regional Norway RHT, slightly more often male, and had a higher level of education than the general population of Norwegian ambulance employees.

**Table 2:** Description of the sample, *N* = 114

		Study sample *N* = 114		All Norwegian ambulance staff, *n* = 5355	
Variable	Categories	n	%	%	
Age group:	18–39	70	61.4	56.7	
	40–54	34	29.8	31.7	
	> 55	10	8.8	11.6	
Regional Health Trust	South-Eastern Norway RHT	52	45.6	47.6	
	Western Norway RHT	18	15.8	14.9	
	Central Regional Norway RHT	26	22.8	16.4	
	Northern Norway RHT	18	15.8	21.1	
Gender	else	0	0	0	
	male	72	63.2	53.5	
	female	42	36.8	46.5	
Education level	bachelor in nursing	26	22.8	n.a.	
	bachelor in paramedic	17	14.9	n.a	
	higher education	43	37.7	28.7	
	lower education	71	62.3	71.3	
Years of experience	< 2	11	9.6	n.a	
	3–9	36	31.6	n.a	
	10–20	42	36.8	n.a	
	> 20	25	21.9	n.a	

n.a. = The level of education and years of experience were not available data for the ambulance staff population in Norway

The mean total sum scores for participants’ data collected in each of the datasets are shown in Table 3.

**Table 3:** Mean sum scores and standard deviation for each dataset (*N* = 114)

When participants chose more than one answer, the answer indicating the most positive regard was chosen				When participants chose more than one answer, the answer indicating the least positive regard was chosen			
Test (dataset 1)		Re-test (dataset 2)		Test (dataset 1)		Re-test (dataset 2)	
Mean	Standard Deviation	Mean	Standard Deviation	Mean	Standard Deviation	Mean	Standard Deviation
47.02	8.2	46.55	8.2	46.6	8.2	46.3	8.2

### Evaluation of measurement properties

#### Floor and ceiling effect assessments

There was a ceiling effect for items two, five, six, seven and eleven. The ceiling effect was strong for items two and seven, where more than two-thirds of the respondents answered the highest possible value on the Likert Scale (70.2% and 71.1%, respectively). For items five, six and eleven, about one in five responders chose the highest possible value (20.2%, 23.7% and 19.3%, respectively). For details, see Table 4. A flooring effect was not found for any of the items.

**Table 4:** The distribution of answers on each item of the Norwegian translation of the Medical Condition Regard Scale

		When participants chose more than one answer, the answer indicating the most positive regard was chosen				When participants chose more than one answer, the answer indicating the least positive regard was chosen			
Item no.	Likert Scale	Test (dataset 1)		Re-test (dataset 2)		Test (dataset 1)		Re-test (dataset 2)	
		n	%	n	%	n	%	n	%
Item 1	1	9	7.9	5	4.4	9	7.9	5	4.4
	2	30	26.3	23	20.2	31	27.2	24	21.1
	3	18	15.8	16	14	19	16.7	16	14
	4	19	16.7	36	31.6	18	15.8	37	32.5
	5	33	28.9	28	24.6	32	28.1	26	22.8
	6	5	4.4	6	5.3	5	4.4	6	5.3
	Total	114	100	114	100	114	100	114	100
Item 2	1	0	0	1	0.9	0	0	1	0.9
	2	0	0	1	0.9	0	0	1	0.9
	3	1	0.9	0	0	1	0.9	0	0
	4	0	0	1	0.9	1	0.9	1	0.9
	5	33	28.9	41	36	33	28.9	43	37.7
	6	80	70.2*	70	61.4*	79	69.3*	68	59.6*
	Total	114	100	114	100	114	100	114	100
Item 3	1	1	0.9	1	0.9	1	0.9	1	0.9
	2	13	11.4	20	17.5	13	11.4	22	19.3
	3	30	26.3	23	20.2	30	26.3	22	19.3
	4	11	9.6	24	21.1	12	10.5	23	20.2
	5	56	49.1	43	37.7	55	48.2	44	38.6
	6	3	2.6	3	2.6	3	2.6	2	1.8
	Total	114	100	114	100	114	100	114	100
Item 4	1	1	0.9	0	0	1	0.9	0	0
	2	20	17.5	23	20.2	20	17.5	23	20.2
	3	11	9.6	16	14	12	10.5	16	14
	4	26	22.8	25	21.9	28	24.6	25	21.9
	5	48	42.1	37	32.5	45	39.5	38	33.3
	6	8	7	13	11.4	8	7	12	10.5
	Total	114	100	114	100	114	100	114	100
Item 5	1	1	0.9	1	0.9	1	0.9	1	0.9
	2	12	10.5	9	7.9	12	10.5	9	7.9
	3	13	11.4	12	10.5	14	12.3	13	11.4
	4	12	10.5	15	13.2	15	13.2	15	13.2
	5	53	46.5	49	43	49	43	49	43
	6	23	20.2*	28	24.6*	23	20.2*	27	23.7*
	Total	114	100	114	100	114	100	114	100
Item 6	1	4	3.5	4	3.5	5	4.4	4	3.5
	2	12	10.5	16	14	14	12.3	18	15.8
	3	7	6.1	10	8.8	6	5.3	10	8.8
	4	11	9.6	14	12.3	12	10.5	16	14
	5	53	46.5	52	45.6	50	43.9	48	42.1
	6	27	23.7*	18	15.8*	27	23.7*	18	15.8*
	Total	114	100	114	100	114	100	114	100
Item 7	3	1	0.9	1	0.9	1	0.9	1	0.9
	4	0	0	0	0	0	0	1	0.9
	5	32	28.1	39	34.2	33	28.9	38	33.3
	6	81	71.1*	74	64.9*	80	70.2*	74	64.9*
	Total	114	100	114	100	114	100	114	100
Item 8	1	6	5.3	8	7	7	6.1	8	7
	2	40	35.1	31	27.2	40	35.1	31	27.2
	3	17	14.9	17	14.9	18	15.8	17	14.9
	4	12	10.5	17	14.9	12	10.5	17	14.9
	5	30	26.3	33	28.9	28	24.6	33	28.9
	6	9	7.9	8	7	9	7.9	8	7
	Total	114	100	114	100	114	100	114	100
Item 9	1	0	0	1	0.9	0	0	2	1.8
	2	14	12.3	12	10.5	14	12.3	11	9.6
	3	17	14.9	11	9.6	21	18.4	12	10.5
	4	41	36	54	47.4	41	36	54	47.4
	5	40	35.1	34	29.8	37	32.5	33	28.9
	6	2	1.8	2	1.8	1	0.9	2	1.8
	Total	114	100	114	100	114	100	114	100
Item 10	1	6	5.3	5	4.4	6	5.3	5	4.4
	2	28	24.6	30	26.3	29	25.4	30	26.3
	3	18	15.8	15	13.2	18	15.8	15	13.2
	4	21	18.4	27	23.7	20	17.5	27	23.7
	5	31	27.2	30	26.3	31	27.2	30	26.3
	6	10	8.8	7	6.1	10	8.8	7	6.1
	Total	114	100	114	100	114	100	114	100
Item 11	1	5	4.4	7	6.1	5	4.4	7	6.1
	2	21	18.4	26	22.8	22	19.3	26	22.8
	3	19	16.7	17	14.9	20	17.5	17	14.9
	4	21	18.4	12	10.5	19	16.7	14	12.3
	5	26	22.8	38	33.3	27	23.7	37	32.5
	6	22	19.3*	14	12.3	21	18.4*	13	11.4
	Total	114	100	114	100	114	100	114	100

* Ceiling effect was found (> 15% of respondents answered the highest possible score on an item)

#### Internal consistency for the items

Cronbach’s Alpha test showed good internal consistency for the items (see Table 5), indicating that the items investigated the same construct [23]. 

**Table 5:** Cronbach’s alpha’s test for the four datasets, *N* = 114

	Dataset	Cronbach’s Alpha	Cronbach’s Alpha Based on Standardized Items
When participants chose more than one answer, the answer indicating the most positive regard was chosen	Test (dataset 1)	0.820	0.819
	Re-test (dataset 2)	0.822	0.824
When participants chose more than one answer, the answer indicating the least positive regard was chosen	Test (dataset 1)	0.821	0.820
	Re-Test (dataset 2)	0.821	0.823

#### Test-retest assessment

Our data did not reveal any statistically significant differences in the total sum score between the first and second data collection (D1 and D2). However, for item one, there was a significantly lower score in D2. Items two and three were not answered significantly differently, but there was a trend toward a higher score in D2 (see Table 6). We arrived at the same conclusion using both pairs of datasets, e.g. the imputation of either low or high values did not change the results.

**Table 6:** Results from the paired sample T-test, investigating differences in the respondents’ answers for each item in dataset 1 (test) and dataset 2 (re-test), *N* = 114

	Item	Mean	95% Confidence Interval of the Difference		*p*-values
			Lower	Upper	
When participants chose more than one answer, the answer indicating the most positive regard was chosen	Item 1	-0.22	-0.43	-0.013	0.037*
	Item 2	0.14	-0.00	0.28	0.055
	Item 3	0.18	-0.03	0.38	0.096
	Item 4	0.08	-0.12	0.28	0.427
	Item 5	-0.11	-0.30	0.07	0.219
	Item 6	0.26	-0.06	0.59	0.109
	Item 7	0.06	-0.04	0.16	0.225
	Item 8	-0.11	-0.38	0.15	0.395
	Item 9	-0.01	-0.19	0.17	0.923
	Item 10	0.04	-0.15	0.24	0.657
	Item 11	0.16	-0.09	0.41	0.213
	SUM scores	0.47	-0.53	1.46	0.356
When participants chose more than one answer, the answer indicating the least positive regard was chosen	Item 1	-0.22	-0.41	-0.03	0.026*
	Item 2	0.14	-0.01	0.29	0.066
	Item 3	0.20	-0.01	0.41	0.061
	Item 4	0.05	-0.15	0.25	0.606
	Item 5	-0.13	-0.32	0.06	0.174
	Item 6	0.25	-0.07	0.58	0.129
	Item 7	0.06	-0.04	0.16	0.238
	Item 8	-0.17	-0.42	0.09	0.203
	Item 9	-0.06	-0.23	0.11	0.470
	Item 10	0.03	-0.17	0.22	0.793
	Item 11	0.15	-0.10	0.40	0.239
	SUM scores	-0.31	-1.28	0.67	0.535

Our sensitivity analysis confirmed (a) a significant difference in how respondents answered item one and (b) that item two had a tendency towards statistical significance but did not reach the significance threshold level.

### Construct analysis

Exploratory and confirmatory factor analyses were conducted to assess possible underlying factors among the items. Exploratory factor analysis was conducted to assess how many possible underlying factors there were in the scale. According to the screen plot, our data did not reveal any underlying structure, and all items loaded only on one factor; thus, our analysis suggested that the questionnaire should be used as a one-dimensional instrument. To our knowledge, no other questionnaire or qualitative data could be used to assess construct validity.

A confirmatory factor analysis with two factors was also conducted. To our knowledge, only one paper has previously reported doing a confirmatory factor analysis on the MCRS on a population of paramedic students [24]. We did not find the same items to load on the two factors identified in our data. Moreover, only 49.3% of the variance was explained by two factors. Therefore, we suggest the scale should be used as a one-dimensional instrument in a Norwegian setting in ambulance staff populations.

## Discussion

The Norwegian translation of the Medical Condition Regard Scale (MCRS) underwent testing in a representative sample of Norwegian ambulance staff. To our knowledge, this is the first time the scale has been translated into Norwegian and rigorously evaluated for its psychometric properties. Our findings indicate that the Norwegian version demonstrates adequate internal consistency and reliability over four weeks. However, it tends to have a ceiling effect on five items limiting the ability to discern differences among high-scoring individuals.

The mean MCRS scores for Norwegian ambulance staff about patients with psychosis ranged between 46 and 47 (depending on the dataset used). In comparison, Christison, Haviland and Riggs et al. [14] reported a mean score of 44.4 for physicians toward patients with mental health conditions. Conversely, for somatic conditions like pneumococcal pneumonia, acute meningitis, or heartburn, they reported values above 50. Physicians’ mean score for patients with frequent visits and diverse symptoms but few physical findings was below 40. Similarly, Norwegian ambulance staff scored patients with psychosis in the mid-range, consistent with Christison, Haviland and Riggs et al. [14] findings.

Health professionals’ attitudes toward medical conditions vary across different professional groups. For instance, Gilchrist, Moskalewicz et al. [25] found that nurses and social workers scored lower on the Medical Condition Regard Scale (MCRS) than physicians and psychologists. To our knowledge, only one study has investigated ambulance staff’s medical condition regards, focusing on paramedic students [24]. The study revealed relatively poor attitudes toward four stigmatised medical conditions, with a mean value of 38.06 reported for first-year paramedic students. The authors emphasise the importance of empathy awareness in improving patient outcomes and recommend targeted education curricula within paramedic degree programs to enhance empathy toward stigmatised patient groups, including those with psychosis.

In the study by Christison, Haviland, and Riggs et al. [14] the test-retest reliability of the original instrument was assessed over a 17-day period. The reported reliability coefficient was 0.84, indicating stability. However, there is no information regarding the reliability of individual items within the instrument. The Norwegian translation of the instrument was also examined. A paired sample T-test revealed no significant differences between the sum scores at two time points (D1 and D2), except for item one. This suggests that the construct being measured remains consistent over time, although there may be isolated issues with specific items. Because the reliability of each item is not reported by Christison, Haviland and Riggs et al. [14] we cannot determine whether the significant difference observed for item one is specific to the Norwegian translation within the ambulance staff population or if it also exists in the original version.

A ceiling effect indicates that a large proportion of respondents have chosen the highest scores on an assessment. This lack of variability poses challenges for internal consistency reliability and subgroup identification. Christison, Haviland and Riggs et al. [14] noted the MCRS’s vulnerability to this effect in the original English version but didn’t specify affected items or how strong the effect was. In the Norwegian translation, items two and seven had a strong ceiling effect. These items, related to insurance coverage and medical expenditures, underwent significant changes during cultural adaptation. Norwegians are used to the Scandinavian welfare system, and it is strongly embedded in the Norwegian culture that health services should be free of charge and equitably available to all. We believe the ceiling effect of items two and seven reflects this cultural phenomenon. This may indicate that cultural adaptation was not sufficient.

The prehospital work environment is generally poorly understood by non-ambulance staff [26]. Thus, a scale that had been developed for other healthcare contexts is not necessarily suitable for prehospital environments. During the cognitive debriefing phase of the translation process, ambulance staff mentioned that items five, six, and eight were unclear in terms of how they were to be answered in a prehospital context. The comments from the cognitive debriefing, which we have not mitigated in the Norwegian translation (only item six was adjusted according to the cognitive debriefing), we believe highlight weaknesses of the original instrument which is also present in the Norwegian translation.

A Norwegian translation of this questionnaire enables us to reliably explore attitudes towards patients with psychosis in an ambulance context. By doing so, we can monitor the effect of education and other competence-enhancement initiatives on attitudes. Patients with mental health conditions report experiencing negative attitudes among healthcare workers [13]. Although their experiences are valuable and must be taken seriously, it is also necessary to measure attitudes from other perspectives than the patient’s perceptions. By using this scale to investigate medical regards, we can tailor education and competence enhancement and improve the quality of services. As stigma can lead to less help-seeking behaviour from patients [12] and more avoidant clinical behaviour among healthcare workers [14], finding effective measures to mitigate negative attitudes and stigma has the potential to increase the quality of services. Consequently, less negative attitudes among healthcare workers may lead to better health for stigmatised patient groups, such as people experiencing psychoses [27]. 

### Strengths and limitations

The study’s main strengths are the large sample size and the sample’s similarity to the general Norwegian ambulance staff population’s characteristics. Consequently, we consider our sample as representative, with no evidence of selection bias. Participants were recruited using open Facebook pages and closed Facebook groups. The groups and pages had many followers/members from all regions of Norway and followers/members with various educations, backgrounds, and levels of experience. Acquaintances and colleagues were used to spread the invitation broadly in our extended network. Consequently, we recruited participants with varied backgrounds and characteristics beyond those from our own network.

Uncertainties exist regarding the proportion of AST and BP in ambulance services due to a national change in healthcare personnel licensing in 2022, and following that, some BPs have been categorised as AST by Statistics Norway for the years between 2017 and 2022. However, given the few paramedic bachelor programs in Norway, we were able to calculate the number of licensed paramedics. Consequently, we believe that the education level among ambulance staff in Norway, as presented in our study, is reasonably accurate. Physicians employed in ambulance services (*n* = 127) are not included in the calculation of the total 5355 ambulance staff, nor are apprentices without completed degrees (*n* = 272). Additionally, Statistics Norway reports 573 individuals with other types of education working in ambulance services, likely in administrative roles. These individuals are reported as ‘ambulance staff’ in this article.

The obvious weakness of this study is that the online surveys, by mistake, allowed participants to provide multiple answers on the Likert Scale for each item. We addressed this issue by conducting sensitivity analyses, which used datasets reflecting both their most positive and least positive regards for ambiguous items with multiple answers. The results were consistent.

## Conclusion

The Norwegian translation of the MCRS demonstrates adequate internal consistency and reliability over time. However, it tends to have a ceiling effect on some items. Our study found that Norwegian ambulance staff’s regard toward patients with psychosis is mid-range, slightly more positive than literature-reported attitudes toward patients with mental health issues in general. Further research is necessary to explore and investigate stigmatisation and negative attitudes among ambulance staff, particularly towards vulnerable patient groups. Our validation of the Norwegian MCRS makes it possible to explore this phenomenon further.

## Data Availability

The datasets used and/or analyzed during the current study are available from the corresponding author upon reasonable request.

## Abbreviations

CRC: Carl robert christiansen
KH: Kristin häikiö
EMEF: Eva marie flaathen
RK: Rune kveen
MH: Milada hagen
